# A case of sitosterolaemia with stomatocyticanaemia and thrombocytopenia treated with Ezetimibe with good response

**DOI:** 10.1186/1687-9856-2013-S1-P174

**Published:** 2013-10-03

**Authors:** Ching-ngar Hung, Ching-yin Lee

**Affiliations:** 1 Department of Paediatrics and Adolescent Medicine, Princess Margaret Hospital, Hong Kong, SAR; 2 Department of Paediatrics and Adolescent Medicine, Caritas Medical Center, Hong Kong, SAR

## 

Sitosterolaemia is a rare autosomal recessive lipid disorder characterized by increased absorption of plant sterols in the gut and decreased biliary excretion of sterols causing accumulation of plasma sterols, which can lead to premature atherosclerosis.

Here we reported a boy presented with multiple tuberous xanthomas at 4 year sold and was diagnosed sitosterolaemia [1]. The fasting plasma total cholesterol and low-density lipoprotein (LDL) cholesterol levels were 18.3 mmol/L and 16.41 mmol/L respectively. Gas chromatography and mass spectrometry showed thatthefasting plasma sterols contained elevated beta-sitosterol 880 µmol/L (Reference range<12 µmol/L), campesterol489 µmol/L (Reference range <17.5 µmol/L) and stigmasterol38.9 µmol/L (Reference range <3.5 µmol/L).Molecular study identified compound heterozygous mutations (R419H and IVS12+IG→A)in the adenosine triphosphate (ATP) binding cassette subfamily G, member 5 (*ABCG5*) gene.

Initial management included dietary restriction in cholesterol and plant sterols and cholestyraminetreatment.The total cholesterol and LDL cholesterol levels decreased.

The boy developed bleeding tendency with gum bleeding and epistaxis and hepatosplenomegalyat 7 years old. Blood test confirmed and thrombocytopenia and peripheralsmear revealed stomatocytichaemolyticanaemia and giant platelets [2]. Bone marrow study showed hypercellular marrow. He was treated with increasing dose of cholestyraminebut the drug compliance was fair.The haematological problems persisted.

At the age of 13,Ezetimibe 10mg daily was added. Ezetimibe blocks the absorption of dietary and biliary sources of cholesterol and plant sterols. The platelet count rose from 58 x 10^9^/L to 107 x 10^9^/L in 4 weeks’ time and normalized after 10 months of Ezetimibe treatment. The haemoglobin level rose from 11 g/dL to 13.8 g/dL in 4 months. The plant sterol level also showed significant improvement (see table 1). There were decreased liver and spleen size. The drug was well tolerated with no adverse effect. The efficacy of Ezetimibe in our patient was sustained after 4 years of treatment which was consistent with the other studies on long-term Ezetimibe treatment [3-6].

In conclusion, Ezetimibe treatment was effective in lowering the plasma cholesterol and sterols level in our patient with sitosterolaemia. It is also effective in reversing the stomatolyticanaemia and thrombocytopenia.

**Table 1:** 

	2004	2006	Mar 2008	Jul 2008 (Ezetimide started in Jun 2008)	Nov 2008	2009	2010	2011
Haemoglobin (13-17 g/dL)	8.8	8.9	11	10.5	13.8	14.6	14.4	15.7

Platelet (150-400 x10^9^/L)	60	59	58	107	100	167	262	124

Campesterol (<17.5 µmol/L)	489	329	266	-	-	199	196	192

Stigmasterol (<3 µmol/L)	38.9	31	19	-	-	22.3	24.8	27.6

Beta-sitosterol (<12 µmol/L)	880	548	617	-	-	443	345	360
